# A novel gel liner system with embedded electrodes for use with upper limb myoelectric prostheses

**DOI:** 10.1371/journal.pone.0198934

**Published:** 2018-06-18

**Authors:** Timothy Reissman, Elizabeth Halsne, Robert Lipschutz, Laura Miller, Todd Kuiken

**Affiliations:** 1 Center for Bionic Medicine, Rehabilitation Institute of Chicago, Chicago, IL, United States of America; 2 Department of Biomedical Engineering, Northwestern University, Chicago, IL, United States of America; University of Illinois at Urbana-Champaign, UNITED STATES

## Abstract

We present the development and evaluation of a gel liner system for upper limb prosthesis users that enables acquisition of electromyographic (myoelectric) control signals through embedded electrodes and flexible, conductive fabric leads. This liner system is constructed using a manufacturing approach rather than by modifying a commercially available liner. To evaluate the efficacy, eight male individuals with transhumeral amputations used this system, with standard myoelectric prostheses, for home trials lasting an average of 7.3 weeks. Before and after the home trials, electrical resistance of the cumulative 218 embedded electrodes and leads within 10 gel liner systems was measured and found to increase slightly (from an average of 13.4 to 27.5 Ω) after usage. While this increase was statistically significant (p = 0.001), all but one of the final resistance values remained low enough to enable consistent myoelectric control. User impressions were evaluated through a questionnaire comparing the liner prototypes to their own myoelectric prosthesis socket interface. Subjects preferred the liner prototype (p = 0.008) over their own system in the clinical areas of comfort, suspension, function, and, especially, ease of use. These results suggest that this gel liner system is a clinically viable option and that it may offer advantages over current clinical technology for users of upper limb myoelectric prostheses.

## Introduction

For nearly three decades, individuals with amputations have used roll-on elastomeric gel liners, made from a variety of materials, as an interface between their residual limb and a rigid outer socket [1–3]. Such liner systems are reported to offer benefits, including cushioning of soft tissue and bony prominences, protection of the skin from friction caused by relative motion between the limb and socket, improved suspension via locking mechanisms or suction sealing, and increased adjustability of fit [3]. Although liners have historically been used for individuals with lower limb amputations, they have also been increasingly adopted for upper limb prosthetic fittings due to their comfort, ease of donning/doffing, and optimal device suspension [4, 5].

For individuals who use myoelectric, externally powered upper limb prostheses, which are controlled by skin-surface electromyographic (EMG) signals, the socket must fit the residual limb tightly to prevent loss of contact between the electrodes—typically mounted in the socket wall—and the skin covering the residual limb muscles. This tight fit often necessitates the use of a donning aid to pull the residual limb tissues into the socket, which has the tendency to make the donning process time-consuming, physically demanding, and prone to error or discomfort. In order to address these challenges, numerous attempts have been made to extend the benefits of a gel liner interface to upper limb myoelectric prosthesis users [6–10].

The fundamental obstacle to using liner systems with myoelectric devices is that the liner prevents the necessary contact between the user’s skin and the socket-mounted electrodes. Attempts to overcome this issue have generally involved modifying existing liners to allow access of electrodes to the skin, either by piercing the liner with metal electrode domes [2, 7, 8] or cutting holes in the liner to expose the skin [6]. While both approaches are functional, they frequently result in damage to the integrity of the liner, reducing its lifespan. Cutting holes in liners to allow skin contact requires users to don the liner in the correct position (i.e., so that the holes line up with the electrodes in the socket) to enable EMG signal transmission. Conversely, while placing domes in the liner removes this requirement, the user must then attach individual wires to each electrode after donning the liner, which is cumbersome and requires the user to manage the wires and ensure proper connections [7]. Additionally, the wires then run between the liner and the socket, so relative movement of liner and socket can potentially cause damage, leading to failure. A third approach to eliminating the wire harness involves pushing metal electrode domes through the liner such that the back of the electrodes align with magnets that are embedded in the socket and attached to wires [9]. However, while this approach improved donning alignment through magnetic assistance and reduced the need for wire manipulation, movement of the residual limb during muscle contractions frequently disengaged the magnets from the electrodes. Central issues with all of these attempts to modify existing liners were that (1) all posed challenges to the user (e.g., need for wire management, precise donning technique to align with socket); (2) all voided manufacturer warranties on the elastomeric liners; and (3) while somewhat successful in allowing the user to wear the gel liner with a myoelectric prosthesis, all required lengthy customization of the liner and often the hard socket. To date, no commercial gel liners suitable for use with a myoelectric prosthesis are available, and only moderate success has been reported with liner modification approaches.

Despite these barriers, gel liners could enhance the use and function of upper limb myoelectric prostheses by offering superior cushioning and skin protection for improved comfort, ensuring consistent contact between the residual limb skin and electrodes, increasing the adjustability of the socket fit, and providing optimal prosthesis suspension via a distal locking mechanism. Integrating both the electrodes and the wires into the liner would eliminate the burden of wire management, making donning easier for the user. We hypothesized that such a myoelectric liner system would provide users with enhanced comfort and ease of use, while maintaining optimal prosthetic function, and would be advantageous compared to previous solutions by eliminating the need for post-manufacturing modifications, thus reducing clinical fabrication time and preserving the integrity of the liner.

Here we describe the development and clinical evaluation of a novel, integrated myoelectric gel liner system that includes electrode domes and wiring embedded during the manufacturing process [10] and that provides electrical connections to the socket electronics and mechanical suspension via a novel distal magnetic locking connector [11].

## Materials and methods

### Integrated myoelectric gel liner system

**Fig 1:**
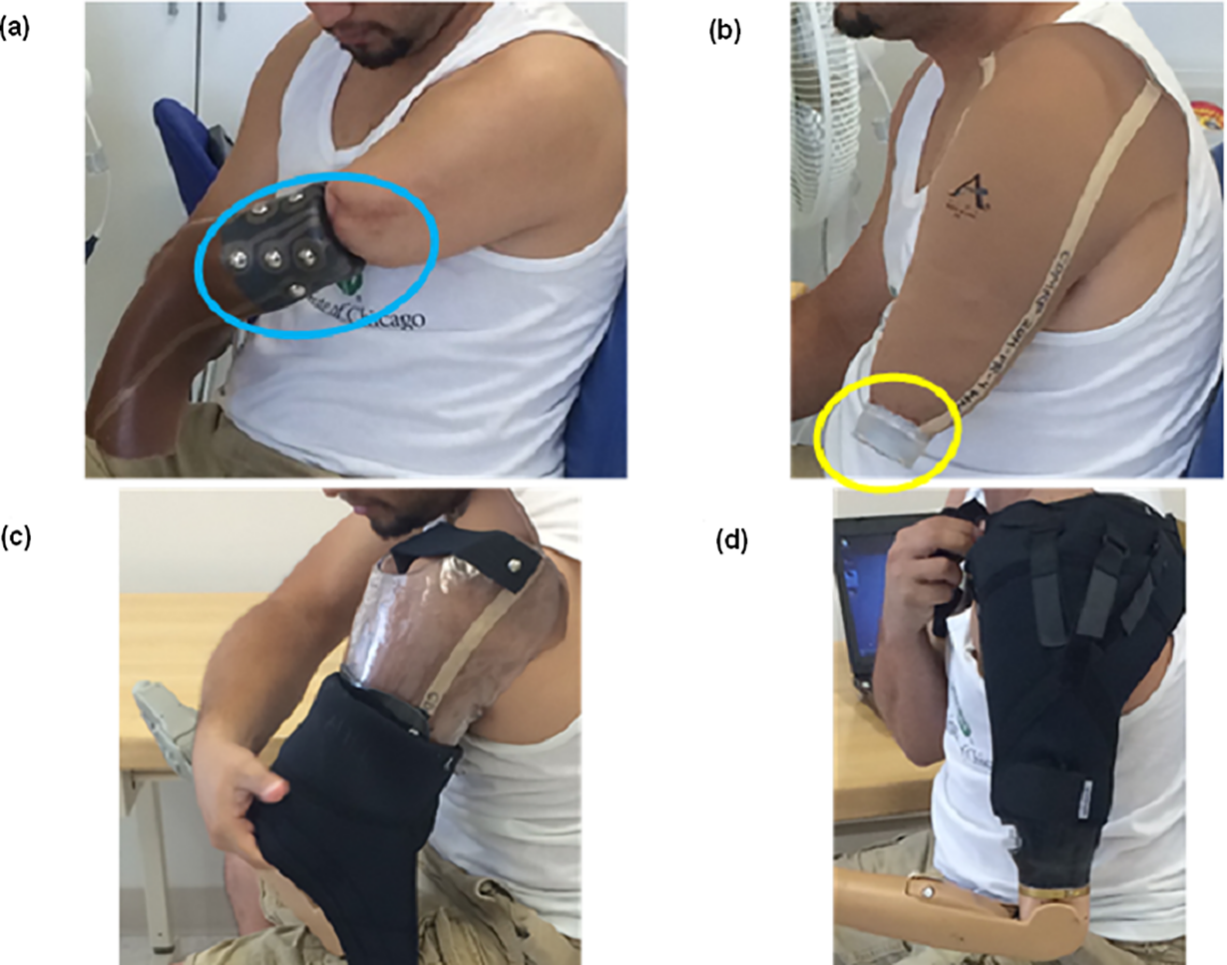
Donning of myoelectric gel liner system. (a) Gel liner system is inverted and then rolled onto the residual limb (A blue oval highlights the electrode domes and the connected fabric leads); (b) Donned liner with proximal magnetic connector at distal end, shown prior to donning myoelectric prosthesis (A yellow oval indicates the proximal connector); (c) Donning of prosthesis with distal magnetic connector embedded at the distal end of the socket. Locking of proximal and distal magnetic connector parts aided by magnetic attraction and secured with cam latch; (d) Securing of myoelectric prosthesis with auxiliary suspension harness.

Commercially available gel liners are made from a range of elastomeric materials (silicone, urethane, thermoplastic elastomers, etc.). The primary concept behind our gel liner system was to integrate the components needed for myoelectric control—i.e., the electrodes and wires (or leads)—into the gel liner during the manufacturing process without compromising the structural or elastic properties of the liner [10, 12]. To achieve this, we encapsulated strips of bi-directional stretch, silver-coated fabric (A321, Less EMF, Latham, NY) between the outer fabric cover of the liner and the gel layer to act as insulated electrical leads. The fabric leads were secured to internally threaded weld nut posts (90611A200, McMaster-Carr, Elmhurst, IL) that were gold plated to prevent corrosion and maintain conductivity; the height of the weld nuts was machined prior to plating to match to the thickness of the gel layer, such that only the top of the weld nuts were exposed on the internal liner surface. Custom stainless steel electrode domes with extended thread rods could then be secured into the weld nuts, exposing the convex dome on the gel surface inside of the liner (Fig 1A) to enable contact with the user’s skin.

**Fig 2:**
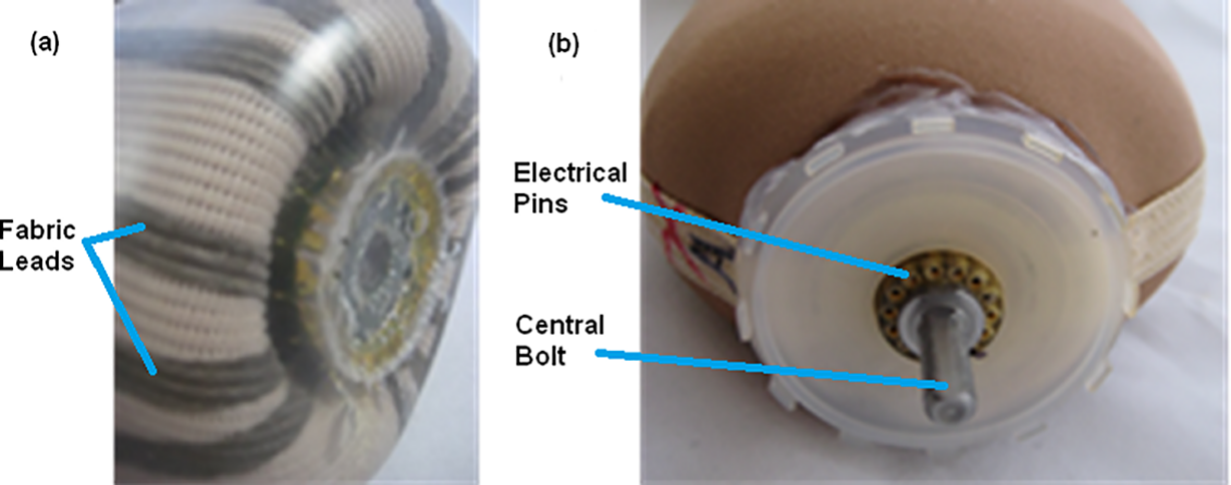
Myoelectric gel liner. (a) Internal view of liner showing central routing of fabric leads; (b) External view of liner in which each fabric lead is electrically connected to hollow electrical pins, and showing the central threaded bolt that the proximal magnetic connector part is secured onto.

The manufacturing process went as follows: (1) conductive silver-coated fabric was cut to form a circular area for an electrode and a contiguous linear extension for the lead; (2) the outer fabric liner cover of the liner (i.e., before the gel layer was added) was turned inside out such that the leads could be attached to the inner side; (3) gold-plated threaded weld nuts were attached to the circular part of the fabric, and the fabric leads and electrodes were attached to the liner cover by ironing with an adhesive (Stitch Witchery, HTC-Retail.com) at desired electrode locations. Since the weld nuts were rigid components in an otherwise flexible system, they were additionally anchored to the liner cover fabric with sewing thread to ensure a secure connection; (4) at the distal end of the liner, the fabric leads were then attached to a custom, central electrical routing connector, which paired each lead with a corresponding conductive hollow pin to allow transmission of EMG signals from the inside to the outside of the liner. These hollow pins formed a perimeter of external connections which were concentric to the distal, internal thread commonly used for securing a locking pin (Fig 2); (5) thermoplastic elastomeric gel was then injected on the inside surface of the liner fabric, according to standard manufacturing practice, covering the conductive fabric leads while leaving the top surface of the weld nuts exposed; (6) stainless steel domes were then threaded into the weld nuts and secured with a thread locking adhesive (Blue 242, Loctite Inc., Westlake, OH), which was non-conductive but did not alter the resistivity of the electrode dome and fabric lead conductive path. The stainless steel domes were used to create an electrode interface that could tolerate the perspiration and abrasion associated with prolonged contact with the skin of a residual limb. The domes were machined to be rounded near the base edges—to minimize any damage to the gel layer—and polished to reduce any skin irritation.

**Fig 3:**
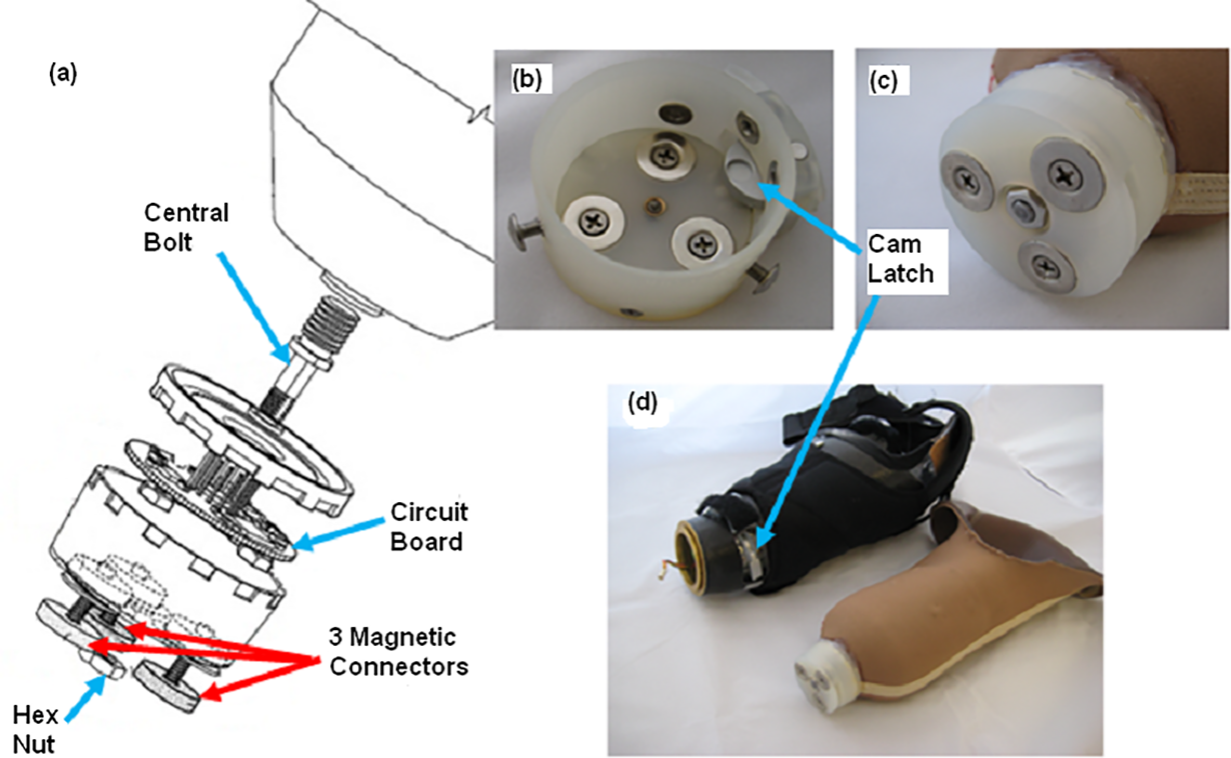
Magnetic electrical connector. (a) Exploded-view drawing of proximal magnetic connector part from [11]; (b) Proximal magnetic connector which is secured to outside of liner; (c) Distal magnetic connector which is secured within distal end of socket; (d) Complete system with proximal connector attached to gel liner and distal connector attached to socket.

A novel magnetic connector interface was designed to simplify mechanical and electrical connection of the liner to the myoelectric prosthesis (Figs 1B and 3). The magnetic connector consisted of two parts: (1) a proximal connector that was attached to the distal end of the liner and (2) a distal connector that was installed inside the distal end of the socket and connected to the myoelectric prosthesis. Three magnets (RA22, K&J Magnetics Inc., Pipersville, PA) incorporated on each connector face served to both assist with mechanical attachment of the two connector parts and to provide electrical connectivity. The magnets were configured such that the two connectors would attract to one another when properly aligned, providing an assistive pull-in force when donning the prosthesis. The force was such that when the two connectors would be placed near one another, the attractive force would nonlinearly increase up to a maximum value of 107N (24lbf) until contact. On each connector face, the magnets were arranged with alternating polarity to assist with proper alignment when donning (i.e., the connector could only be secured when the magnets were in the correct orientation). This proved useful for doffing, as the moment required for magnet separation was only 1.1Nm (10 in-lbf). Relative rotation of the connector interface would increase the distance between the magnet pairs, thereby reducing the attractive force. More relative rotation could bring same polarity magnets together, and the resulting repulsive force could help push the connectors apart. To further secure the connectors axially and prevent relative rotation during use of the prosthesis, a secondary mechanical lock, a cam latch, was used. With this secure and properly aligned connector interface, electrical connections from the liner to the myoelectric prosthesis were ensured by simply securing the cam latch, without the need to manage any wire connections.

The proximal connector was assembled as follows: (1) a threaded bolt extending from the distal end of the liner (Fig 2B) was inserted through the center of the proximal connector and axially secured to the liner by a hex nut (Fig 3C); (2) the perimeter of the lid of the proximal connector was adhered to the outside of the liner using silicone (Fig 2B) to mechanically secure the connector to the liner in case of twisting moments when in use or when doffing; (3) pins from the circuit board housed within the proximal connector (see Fig 3A) were inserted into the hollow pins of the electrical routing connector on the liner. These connected each electrode dome and lead pair to the circuit board in the proximal connector, which served to amplify each EMG signal and perform analog to digital conversion; (4) the circuit board was electrically connected to the three magnets and the center threaded bolt. After conditioning the EMG signals, the circuit board transmitted the high and low signal pathways to the attached distal connector unit using a standard controller area network bus for communications, via two of the three external magnets. The two remaining conduction pathways, the third magnet and central bolt, were used to supply power and signal ground, respectively, to the circuit board.

The distal connector was assembled as follows (Fig 3B): (1) a designated space was formed, using a dummy, at the distal end of the socket to create a cylindrical housing for the distal connector (Fig 3D). Holes were made in the socket for mechanical attachment of the three external set screws and positioning of the mechanical latch. (2) magnets were configured such that when correctly aligned with the proximal connector, the magnets would attract the two connector parts into proper alignment, enabling operation of the mechanical latch and ensuring correct electrical connections; (3) the distal connector was secured by three set screws that secured into internally threaded posts within the wall of the distal connector; (4) the mechanical latch unit was secured onto the distal connector using additional set screws; (5) communication lines, including the power and ground, between the distal connector and the myoelectric prosthesis controller were connected through magnet pairings, and one of two audio signals was provided to indicate if proper connection was made.

The final system was a flexible, elastic gel liner system with integrated EMG sensors, lead wires, and a simple-to-use electrical and mechanical connector. Although initial attempts at using silver conductive fabric for the electrodes as well as for the leads resulted in successful EMG acquisition during early lab testing [12], electrical resistance measurements revealed that prolonged use of the silver-coated fabric as the electrode contact surface led to corrosion of the silver in the fabric, due to the combined effects of perspiration and skin abrasion, and subsequent loss of myoelectric signal conduction, i.e., open circuit conditions [13]. Subsequent testing of liners with stainless steel electrode domes confirmed that this evolved system design was a robust means for collecting EMG signals, over prolonged use. Thus we evaluated our liner system, with stainless steel dome electrodes, in the following clinical study.

### Gel liner system evaluation

The study presented within this work was approved by the Institutional Review Board at Northwestern University, and all subjects provided written consent prior to the study. Prototype socket interface systems comprising gel liners with embedded electrodes, conductive fabric leads, and a magnetic connector interface were evaluated by individuals with unilateral transhumeral amputations who were current myoelectric prostheses users (Table 1). Subject recruitment for this evaluation coincided with the individuals’ participation in a separate, ongoing study in which they used a standardized transhumeral myoelectric prosthesis after having undergone targeted muscle reinnervation surgery [14]. The protocol for this other study required two home trials using different myoelectric control systems (direct control which used 9 subject specific electrode locations and pattern recognition control which used 13 grid based electrode locations). Thus subjects each wore the two separate patterns of the domes within their liner systems to test each control strategy—one dome pattern of the liner for one home trial and the other dome pattern for the second home trial. Prior to and immediately after each home trial, separate evaluations for this study were concurrently performed with the coinciding myoelectric control study. It should be noted that for the other study, the selection of which control strategy first used was randomized, so Liners 1 and 2 within Table 1 are also randomized and thus simply indicate whether the gel liner system was used in the first or second home trial respectively. The liner system was evaluated over the course of prototype development. As such, some of the subjects used an earlier version (conductive fabric electrode domes) for part of their study, as noted in Table 1. However, all subjects also used the final design, described in detail above, in which stainless steel electrode domes were secured to embedded gold-plated weld nut posts and conductive silver fabric leads in the liner. Only results from the final liner design are analyzed in this study. It should be noted that Subject 8 used the same final version liner that was used by Subject 2, see Table 1.

**Table 1:** Subject demographics.

Subject ID	Age*	Side of amputation	Years since amputation*	Weeks at home–Liner 1	Weeks at home–Liner 2	Terminal Device used during study***	Device used at home
**1**	45	Right	2	8**	8	Otto Bock Variplus speed hand	Dynamic Arm, Otto Bock Variplus speed hand
**2**	54	Left	6	10**	8	ETD with wrist flexion	Boston Arm, ETD
**3**	58	Left	5	7**	8	ETD with wrist flexion	Boston Arm, WR, ETD
**4**	25	Left	6	7	6	ETD with wrist flexion	Utah Arm, WR, i-limb Hand
**5**	31	Left	8	6	11	Greiffer	Boston Arm, WR, i-limb Hand
**6**	27	Right	2	7	7	Greiffer	Dynamic Arm, Otto Bock Variplus speed hand
**7**	31	Right	1	6**	6	ETD with wrist flexion	Utah Arm, i-limb Hand
**8**	35	Right	4	7**	6	ETD with wrist flexion	Dynamic Arm, WR, ETD

ETD = Electronic Terminal Device from Motion Control, WR = Wrist Rotator

* At time of consent

** Prior liner system used with silver conductive fabric for the electrode domes

*** Included Boston Digital Arm and a Motion Control wrist rotator

Subjects were fit with a standardized transhumeral myoelectric prosthesis, which included a laminated socket with a Boston Digital Arm System elbow (Liberating Technologies, Inc.; Holliston, MA), a powered wrist rotator, and their personal terminal device—whichever powered hook or hand that they preferred to use with their normal prosthesis (Table 1). The prototype gel liner system was used as an interface inside of the socket, and the magnetic connector served as the primary prosthesis suspension method. Auxiliary suspension of the prosthesis was provided by a standardized harness (AcrocomforT Shoulder Support; Otto Bock, Duderstadt, Germany). The magnetic connector interface also served as the electrical connection between the embedded electrodes and the prosthesis, and contained electronics to amplify and process the collected EMG information.

Subjects were instructed to use the gel liner system with their prosthesis over a period of approximately eight weeks at home and to return to the laboratory for evaluation. Each subject then repeated the same protocol a second time with a different control system, requiring another liner, with the same prosthesis. Prior to using each gel liner system at home, subjects were given five consecutive days of training and use in the laboratory with a prosthetist and occupational therapist to become familiar with the control system. Training with the prosthetist and occupational therapist occurred in tandem, with modifications made to the socket and control as needed by the prosthetist as the subject progressed through therapy and various tasks. In general, subjects were working with the prosthesis four to six hours on each day as fatigue would allow. Subjects were also trained to be able to independently and reliably don and doff the liner system and prosthesis. Directly before and immediately after home use, the electrical resistance of the embedded electrodes and leads within the liners were measured with a multimeter (110 True RMS Digital Multimeter; Fluke Corporation, Everett, WA); these results were used to inform development of the gel liner system (the measurements collected can be found in the S1 File). The stability of the system’s electrical conductivity was monitored because high electrical resistance (above 200 Ω threshold), and its corresponding reduced signal to noise ratio, results in a degradation in the ability to effectively use myoelectric control [15, 16]. A paired-sample t-test analysis was performed to determine statistical significance of any changes in resistance with respect to before and after home trials (p<0.05).

To quantify clinical efficacy, user impressions were obtained through a subjective socket comfort score using an 11 point scale (0–10), where 0 represents the most uncomfortable and 10 represents the most comfortable fit [17]. Subjects were asked prior to their home trials to score their hard socket interface. Subjects were then asked following both home trials to score the liner system. A nonparametric Mann Whitney U test was performed to compare socket comfort scores (statistical significance p<0.05). More detailed user input on comfort, ease of use, suspension, and prosthetic function while using the gel liner system was obtained through a custom questionnaire that also asked subjects to compare the final version of the liner system to their home socket interface. The questions asked in each clinical area are listed in the S2 File. Subjects recorded their perceptions of the liner system by indicating the degree of their agreement with each survey item using a 5-point Likert scale. A nonparametric Wilcoxon signed rank test was performed to determine statistical significance (p<0.05).

## Results

Table 1 represents the demographic and study information for the eight subjects who participated in the study. All subjects were male and wore skin fit sockets with auxiliary harnesses for their devices used at home. Subjects 1, 2, 3, 7, and 8 were the first to conduct the study and used both the prior liner design (conductive fabric electrode domes) and the final design (stainless steel electrode domes). These subjects used the prior version for an average home trial time of 7.6 weeks per person; average electrode electrical resistance before home trials was 17.4 ±9.2Ω (average resistance ±standard deviation) and was 152.0 ±139.7Ω after home trials. Because the electrical resistance after use often exceeded the pre-amplification thresholds of approximately 200 Ω, poor myoelectric control was the result. At this point during the course of the study, the liner design was modified to incorporate stainless steel electrodes in order to stabilize the electrical resistance. Only the results pertaining to the electrical resistances of the final system are included in the results, and subjects were instructed to answer the questionnaire based on their experiences with this final liner system only.

**Fig 4:**
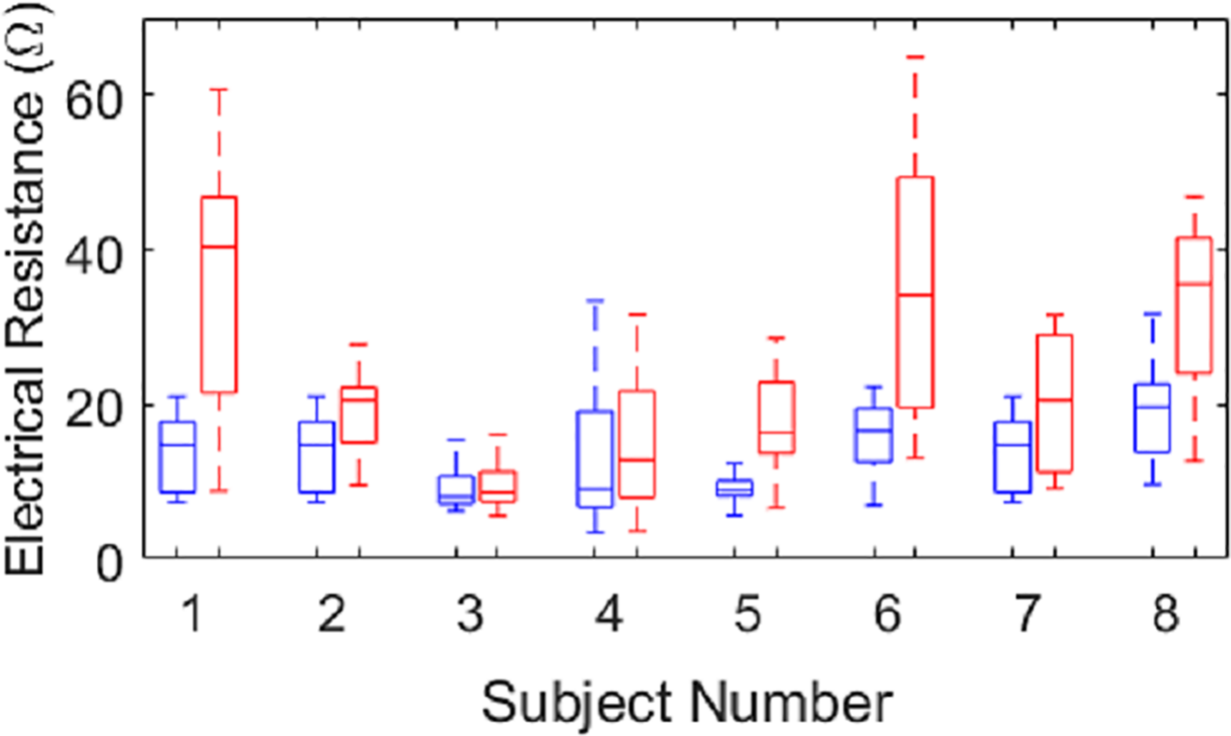
Electrical resistance of gel liner system before (blue) and after (red) home trials for all eight subjects. Note that values under 200 Ω pre-amplification threshold yield useful signal-to-noise ratios for consistent myolectric control.

The average home trial time per person for the final liner design was 7.3 weeks, with a maximum of 11 weeks (Subject 5). The average electrical resistance before home trials was 13.4 ±5.9 Ω (Fig 4). An increase in resistance after home trials was seen for all eight subjects who used the liner system. Although none of the stainless steel electrode dome and insulated fabric lead combinations reached open circuit conditions, for Subject 6, one electrode from the second home trial liner did reach 500 Ω after use and exhibited some surface corrosion. This poor quality from one signal however did not appear to have an effect on control or perceived functionality, as evident by the still favorable self-report by Subject 6 within the survey results, specifically Questions 12 and 22. The mean resistance after the home trials for the final liner design was 27.5 ±48.8 Ω, which was statistically a significant increase (p = 0.001) but still well within pre-amplification thresholds of 200 Ω for effective myoelectric control.

In terms of the longevity of the liner system, Subject 8 received the same liner for the second home trial as that of Subject 2, see Table 1. The combined home trial testing for this liner system was 14 weeks, with 22 weeks of time in storage between the end of Subject 2’s home trial and the beginning of Subject 8’s home trial. The electrical resistance values before the first and after both trials were measured at 13.9 ±4.6 Ω and 32.7 ±11.1 Ω, respectively. During the storage period, nearly no change in resistance occurred for this liner, with values measured before storage at 18.6 ±5.4 Ω and afterwards at 18.7 ±6.0 Ω. After 22 weeks of storage time for Subject 3’s liner after the second home trial, resistance was 13.7 ±7.7 Ω, compared to 9.2 ±3.9 Ω before storage.

**Fig 5:**
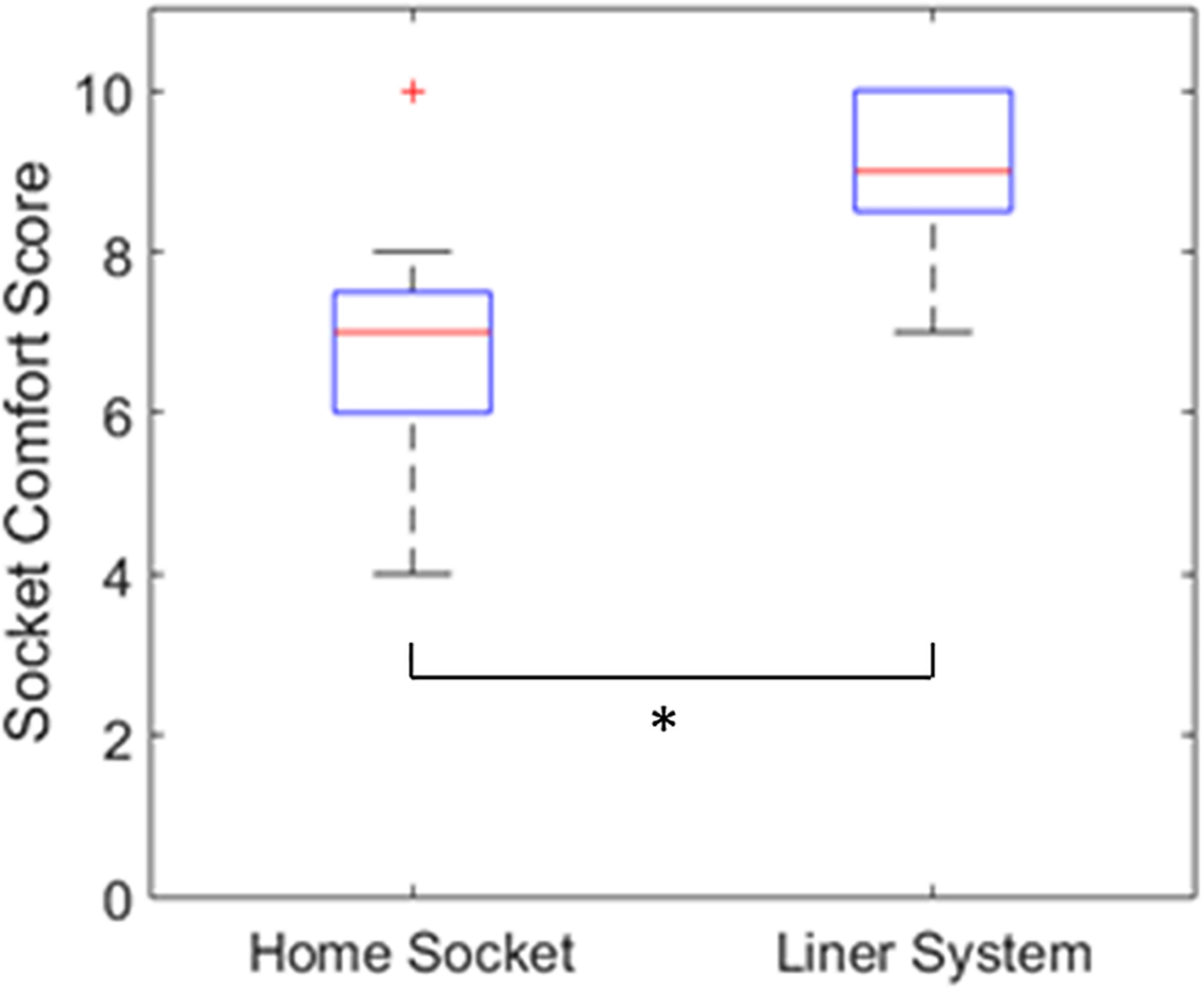
Self-reported socket comfort scores (0–10 range) for the subjects' home sockets and the liner system. Higher scores indicate greater perceived comfort. * Indicates statistical significance (p = 0.02).

Before home trials, subjects indicated their perceived socket comfort scores for their own socket system (Fig 5). Average subject scores for their own sockets were 6.9 ±1.7. Following home trials, subjects scored the liner system. Average subject scores were 9.0 ±1.0; higher scores indicate better comfort. Each subject scored the embedded liner system equal to or better than their own socket, with significant overall differences between the two groups (p = 0.02).

**Fig 6:**
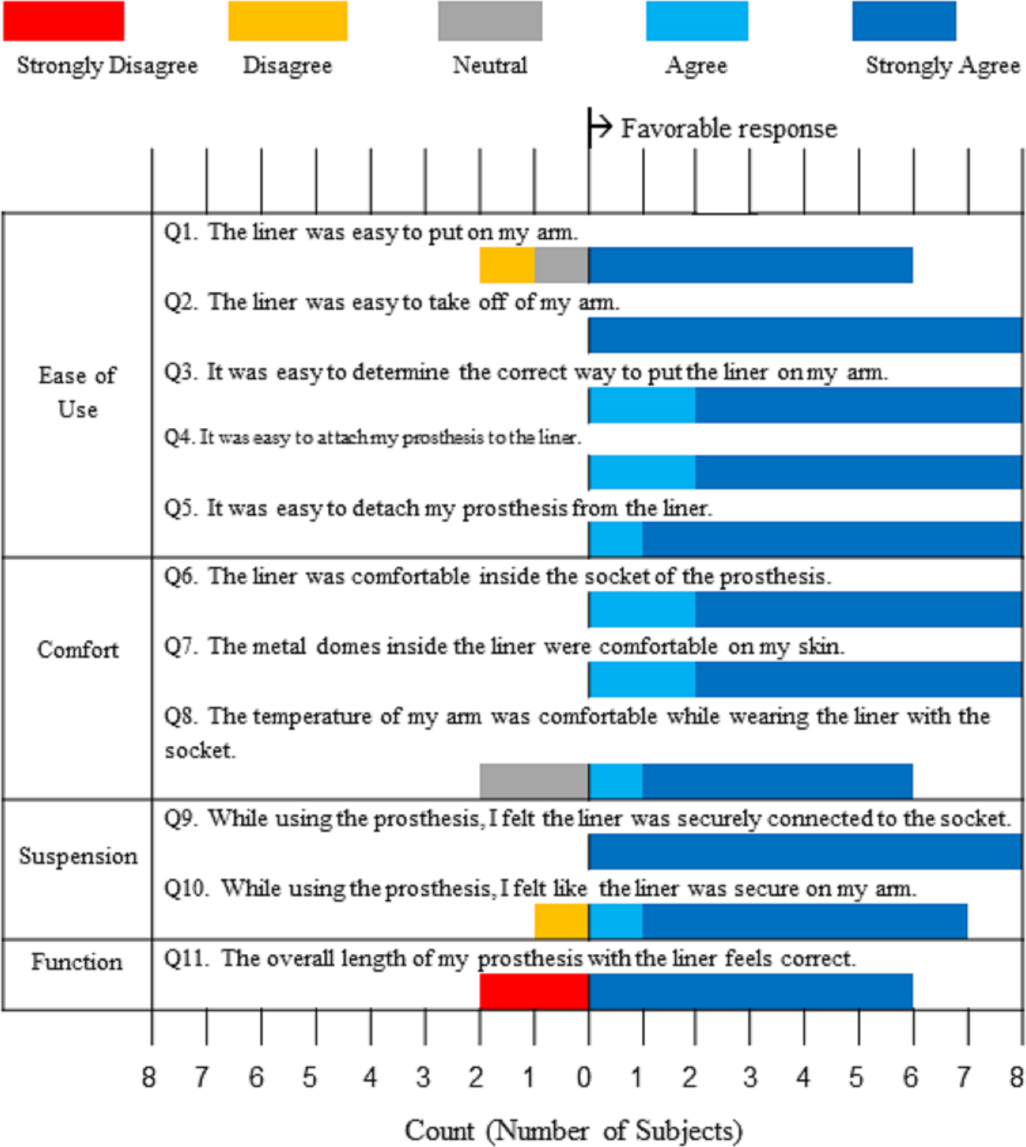
Experience with myoelectric gel liner system. Divergent stacked bar chart structured such that counts to the right indicate a favorable response to the questions within the four clinical areas: ease of use, comfort, suspension, and function. Note: Question 12 and subjects’ responses are included separately in the Results section, as the Likert scale was not used.

Additionally, the custom questionnaire in the S2 File was given to subjects after their second home trial. The first half of the questionnaire consisted of 11 questions based solely on each subject’s experience with the final liner system (Fig 6). Fig 6 is represented graphically using a divergent stacked bar chart [18], which represents favorable responses to the right and neutral/unfavorable responses to the left of the zero count point. Each question was categorized into one of four clinical areas: ease of use, comfort, suspension, and function. Over all the questions, subjects’ answers tended towards favorable answers for the gel liner system in all four categories. The ease of use category had sufficient Likert data to perform a separate Wilcoxon signed rank test, which showed that the favorable response was statistically significant (p = 0.008). The same significance (p = 0.008) was found over all questions for favorable evaluation of the liners over a neutral response.

A single question addressed reliability of the liner system with the magnetic electrical and mechanical connector: Q12. “How frequently did you hear the three-tone failure sound indicating that the liner did not connect to the socket?” Subjects could choose from the following options: never, once a month, once a week, several times per week, once a day, several times per day, or every time I put the prosthesis on. Subject 7 selected never. Subjects 3, 4, and 8 selected once per month. Subjects 1, 2 and 6 selected once per week, and Subject 5 selected several times per week. Thus, the median frequency was between once a month and once a week.

**Fig 7:**
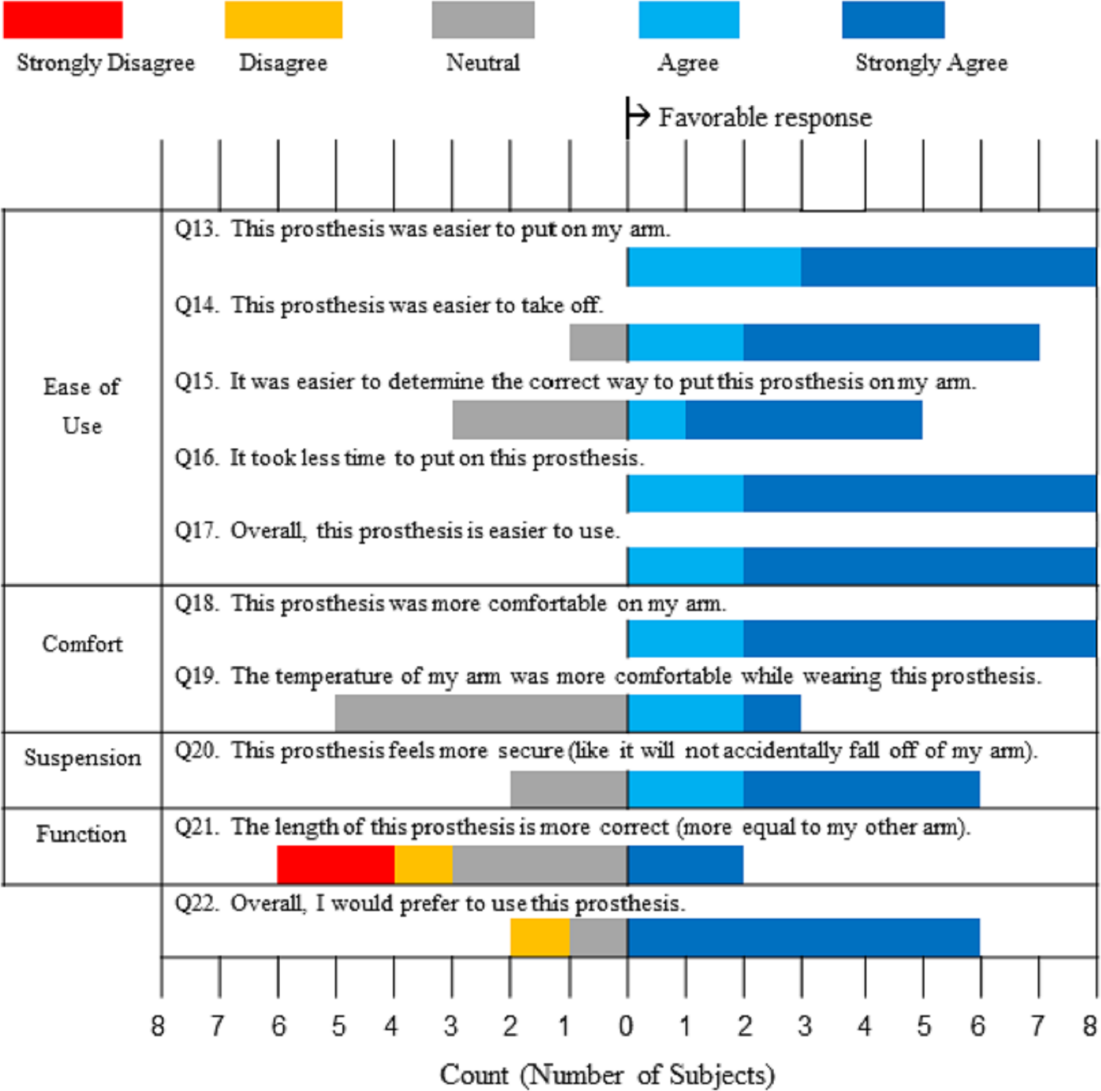
Comparison of myoelectric gel liner system to own system. Divergent stacked bar chart structured such that counts to the right indicate a favorable response to the questions within the four clinical areas: ease of use, comfort, suspension, and function.

The second half of the questionnaire consisted of 10 questions (Fig 7) that asked each subject to compare their experience using the liner system to that using their home system. The same graphical representation used in Fig 6 was applied for Fig 7. For all but one of the questions, a trend emerged that indicated a favorable bias by the subjects towards our liner system over their own systems. The one question that did not follow this trend was Q21, which asked about the length of the prosthesis with the liner. Performing Wilcoxon signed rank tests on the ease of use and on the overall responses, indicated that subjects preferred the gel liner system over their present home systems (p = 0.008).

## Discussion

Our final liner system design with stainless steel electrode domes was stable in terms of electrical resistance, with an average increase in resistance of only 14.1 Ω after a home trial, over the average initial resistance of 13.4 Ω. Additionally, extended use of the same final liner design over two home trial periods, 11 weeks total, showed nearly the same rate of increase in resistance, with a total increase of 18.8 Ω over the initial 13.9 Ω. While noting that the increased resistance after each home trial period was statistically significant, it is important to note that the final resistance values measured remained sufficiently small, well under 200 Ω, to maintain useful signal-to-noise ratios for consistent myoelectric control. As such, the increases in resistance observed did not appear to be functionally significant. Only one of the stainless steel electrode dome and fabric lead pairs exhibited values over 70 Ω after home trial use, and none exhibited open circuit conditions. Thus 99% of all electrode sites maintained values well within the pre-amplification thresholds for effective myoelectric control. In more direct comparison to the improvement over the previous liner design, Subjects 1, 2, 3, 7, and 8 wore the initial liner design with conductive fabric electrodes for one of their home trials within this study. The results showed an average increase in resistance per home trial of 134.6 Ω after the home trial compared to an initial average resistance of 17.4 Ω. As in the previous benchtop study [13], some electrodes even reached infinite resistance or open circuit conditions. These results further indicate that the use of silver-coated fabric electrodes for long-term EMG acquisition is not feasible, as they are susceptible to the corrosive effects of perspiration and abrasion conditions. Thus, using stainless steel domes as the EMG contact surface proved necessary and highly effective at providing stable electrical performance during prolonged use.

The importance of this relatively stable performance can be estimated with respect to the present mean warranty times of 6 to 12 months for prosthetic liners. Applying the approximate rate of 14.1 Ω increase per 7.3 weeks of usage (average increase per trial period), the projected final value after one year of usage is 100.4 Ω over the nominal 13.4 Ω. Thus, even at the end of the liner’s warranty, the electrical performance is projected to be within myoelectric control thresholds and thus functional. As for storage of the liners, examining two liners after 22 weeks of storage showed only a slight increase in mean values of 2.2 Ω over their nominal 13.9 Ω. As such, these liners can likely be stored for long periods of time without degradation of electrical performance. However, as this study is limited in the number of liners tested (N = 10) and the length of usage time, additional testing over longer periods of time is necessary in order to test the accuracy and merit of such projections.

The use of rigid, gold-plated weld nuts and stainless steel electrode domes did not noticeably alter elastic properties of the liner, including bi-directional stretch. No defects were visible to the naked eye in the liner fabric or on the gel layer after the home use trials. Fig 1A displays a picture of the interior of a liner before home use, and Fig 3D shows the exterior of the same liner after home use. Additionally, pictures of the subjects’ skin on their residual limb after using the liner showed only temporary indentation marks, similar to dome imprints found after using their clinical prostheses, and no signs of irritation from the electrode domes were observed.

The magnetic connector interface design proved useful in assisting with donning and doffing of the device. Users informally commented that they could feel the connector pull into place near the end of the donning process and could confirm proper connection and alignment easily with a click sound from the contact of the magnet pairs. Total time observed for subjects to don the liner and prosthesis was approximately 30 seconds, not including securement of the auxiliary harness (an additional 30 seconds). Subjects also commented that with the latch open, the system was easy to doff as they could use the proximal trim lines of the socket as a moment arm to provide the necessary torque to rotate and separate the connector interface, lowering the attractive force via misalignment of the magnet pairs. Total time observed for subjects to doff the liner and prosthesis was approximately 20 seconds, not including detachment of the auxiliary harness.

Analysis of socket comfort scores and questionnaire responses supported our hypothesis that our liner system would enhance comfort and ease of use in comparison to currently available prosthetic interface and suspension systems. In terms of the socket comfort scores, the improvement may have been expected as the subjects received a new socket for the study and had five consecutive days of training with a prosthetist. However, the significant difference in comfort scores between the liner system and subjects’ own systems does indicate a fundamental change in the perceived comfort between the two interfaces. Suspension was shown to also be highly favorable, with only one individual, Subject 5, noting that the liner could have been a size smaller for better fit. It should be noted that this lack of an ideal fit may have contributed to this individual’s high rate of connectivity issues compared to other subjects’ experiences as reflected in the responses to Question 12.

Prosthesis length was increased by addition of the magnetic connector, and subjects had a more varied response to this feature. The connector assembly added approximately 24mm (0.94in) in arm length, so for individuals with long residual limbs this additional length may be problematic.

The following comments were written by the subjects as a part of the questionnaire: “I love the liner”; “There was no problem putting it on”; “I thought the liner was easy and comfortable”; “I like the suspension of the liner”; “It prevents air pockets”; “I like it a lot better than what I have”. Areas in which the liner system scored lower than the users’ own systems on the questionnaire also correlate with users’ written comments—on comfort: “Temperature OK till end of day then sweating”; suspension: “The only change would be a better way of securing a loose fitting liner”; and function: “Make liner connector shorter”. The first two issues are difficult to resolve as they are inherent to the use of gel liners in general. Making the magnetic connector shorter may be a possibility; however as noted previously for individuals with long residual limbs, this issue may continue to be a problem. Additionally, the length of the magnetic connector system in its current configuration is comparable to commercially available upper limb pin-locking liner suspension systems; the Otto Bock 14A1 Upper Extremity Lock Set is 22mm in length.

## Conclusions

Here we present the development and evaluation of a novel gel liner system with embedded electrodes and leads for use with upper limb myoelectric prostheses. Integrating the electrical components within the gel liner during the manufacturing process maintained low electrical resistance even after an average of 7.3 weeks of home usage. Additionally, combining rigid electrodes with flexible fabric leads did not affect the structural integrity or elastomeric properties of the liner during the evaluation period. Subjects perceived the liner system as more favorable than their current system in many areas that are clinically important for a myoelectric prosthesis interface: ease of use, comfort, suspension, and function. In conclusion, we have shown that the benefits of a gel liner system can be provided to myoelectric prosthesis users using a manufactured approach.

## Supporting information

S1 File Study data. Resistivity measurements collected from the 10 gel liner systems using the final design is provided. Additionally data from the socket comfort scores and questionnaires is provided with respect to each subject.
(XLSX)

S2 File Questionnaire used in the study. The original questionnaire given to subjects following their second home trial is provided.
(PDF)
