# Supplementary material for: A novel gel liner system with embedded electrodes for use with upper limb myoelectric prostheses
Source: PLoS One. 2018 Jun 18;13(6):e0198934. doi: 10.1371/journal.pone.0198934 (PMC6005573; doi:10.1371/journal.pone.0198934)
Supplement: S2 File — The original questionnaire given to subjects following their second home trial is provided. (PDF) [file pone.0198934.s002.pdf]

RIC Neural Control Liner Survey

Pages 1-2 to be completed by study representative (prosthetist or OT), not participant.

Subject ID Code: ________

Date of Survey Completion: (home socket eval) ________ (liner/survey) ________

Home Myoelectric Prosthesis: evaluated at initial visit, or another visit when home prosthesis is available

1. Socket Comfort Score: ________

   "On a 0 – 10 scale, if 0 represents the most uncomfortable socket fit you can imagine, and 10 represents the most comfortable socket fit, how would you score the comfort of the socket fit of your artificial limb at the moment?

2. Skin evaluation performed by prosthetist/OT:

   ☐ Photograph skin after doffing (front, side, back, or other relevant areas)

   ☐ Check and document any signs of redness, irritation, other skin issues:

   ____

   ____

   ____

   ____

   ____

   ____

RIC Neural Control Liner: evaluated after post-home trials of both control systems

1. Socket Comfort Score: ________

"On a 0 – 10 scale, if 0 represents the most uncomfortable socket fit you can imagine, and 10 represents the most comfortable socket fit, how would you score the comfort of the socket fit of your artificial limb at the moment?

2. Skin evaluation performed by prosthetist/OT:

   - ☐ Photograph skin after doffing (front, side, back, or other relevant areas)
   - ☐ Check and document any signs of redness, irritation, other skin issues:

     ____

     ____

     ____

     ____

     ____

     ____

Survey Section 1:

Instructions: Please complete the following survey based on your experience with the liner you have been provided for this study. On a scale of 1 to 5, 1 being Strongly Disagree and 5 being Strongly Agree:

EASE OF USE	Strongly Disagree ←		Neutral		→ Strongly Agree
1. The liner was easy to put on my arm.	1	2	3	4	5
2. The liner was easy to take off of my arm.	1	2	3	4	5
3. It was easy to determine the correct way to put the liner on my arm.	1	2	3	4	5
4. It was easy to attach my prosthesis to the liner.	1	2	3	4	5
5. It was easy to detach my prosthesis from the liner.	1	2	3	4	5

COMFORT	Strongly Disagree ←		Neutral		→ Strongly Agree
6. The liner was comfortable inside the socket of the prosthesis.	1	2	3	4	5
7. The metal domes inside the liner were comfortable on my skin.	1	2	3	4	5
8. The temperature of my arm was comfortable while wearing the liner with the socket.	1	2	3	4	5

SUSPENSION	Strongly Disagree ←		Neutral		→ Strongly Agree
9. While using the prosthesis, I felt the liner was securely connected to the **socket**.	1	2	3	4	5
10. While using the prosthesis, I felt like the liner was secure on **my arm**.	1	2	3	4	5

FUNCTION | Strongly Disagree ← Neutral → Strongly Agree

11. The overall length of my prosthesis with the liner feels correct. 1 2 3 4 5

12. How frequently did you hear the three-tone failure sound indicating that the liner did not connect to the socket correctly? (circle one)

0	1	2	3	4	5	6
Never	Once a Month	Once a Week	Several Times per Week	Once a Day	Several Times per Day	Every time I put the prosthesis on

You have the option of providing additional feedback by answering the following questions.

1. Would you like to add any additional comments about the liner (ease of use, comfort, suspension, function, etc)?
____
____
____
____
____
____
____
____

2. What would you change to improve the liner, or the way it is used with the prosthesis?
____
____
____
____
____
____
____
____

Survey Section 2:

Instructions: Please complete the following survey based on your experience with the prosthesis you were given for this study. On a scale of 1 to 5, 1 being Strongly Disagree and 5 being Strongly Agree:

Please **compare** this prosthesis (liner and study prosthesis) to your home myoelectric prosthesis.

EASE OF USE	Strongly Disagree ←		Neutral		→ Strongly Agree
13. This prosthesis was *easier* to put on my arm.	1	2	3	4	5
14. This prosthesis was *easier* to take off.	1	2	3	4	5
15. It was *easier* to determine the correct way to put this prosthesis on my arm.	1	2	3	4	5
16. It took *less* time to put on this prosthesis.	1	2	3	4	5
17. Overall, this prosthesis is *easier* to use.	1	2	3	4	5

COMFORT	Strongly Disagree ←		Neutral		→ Strongly Agree
18. This prosthesis was *more* comfortable on my arm.	1	2	3	4	5
19. The temperature of my arm was *more* comfortable while wearing this prosthesis.	1	2	3	4	5

SUSPENSION	Strongly Disagree ←		Neutral		→ Strongly Agree
20. This prosthesis feels *more* secure (like it will not accidentally fall off of my arm)	1	2	3	4	5

FUNCTION	Strongly Disagree ←		Neutral		→ Strongly Agree
21. The length of this prosthesis is *more* correct (more equal to my other arm).	1	2	3	4	5
22. Overall, I would *prefer* to use this prosthesis.	1	2	3	4	5
